# NK cell immunotherapy after analytic treatment interruption is associated with HIV viral control

**DOI:** 10.1016/j.omta.2026.201778

**Published:** 2026-06-11

**Authors:** Liliana K. Thron, Pongthorn Pumtang-on, Jae-Woong Chang, Maxwell E. Cantor, Ian Gorrell-Brown, Kelsie L. Becklin, Zoe E. Quinn, Ahmad F. Karim, Aaron K. Rendahl, Mary S. Pampusch, Sofia A. Casares, Vaiva Vezys, Branden S. Moriarity, Pamela J. Skinner

**Affiliations:** 1 Department of Veterinary and Biomedical Sciences, University of Minnesota, Minneapolis, MN 55455, USA; 2 Graduate Program in Molecular Pharmacology and Therapeutics, University of Minnesota, Minneapolis, MN 55455, USA; 3 Department of Pediatrics, University of Minnesota, Minneapolis, MN 55455, USA; 4 Agile Vaccines and Therapeutics, Infectious Diseases Directorate, Naval Medical Research Command, Silver Spring, MD, USA; 5 Center for Immunology, Department of Microbiology and Immunology, University of Minnesota, Minneapolis, MN 55455, USA; 6 Masonic Cancer Center, University of Minnesota, Minneapolis, MN 55455, USA; 7 Center for Genome Engineering, University of Minnesota, Minneapolis, MN 55455, USA

**Keywords:** HIV, humanized DRAGA mice, NK cells, CAR therapy, base editor, nonviral engineering, transposon engineering

## Abstract

Over 39.9 million individuals are living with HIV worldwide. There is a need to develop novel therapeutics to improve treatment or cure HIV. In this study, we evaluated HIV reservoir-targeting chimeric antigen receptor (CAR)/CXCR5 natural killer (NK) cells armored with IL-15 and PD-1 knockout as well as control activated NK cells for their safety and efficacy by treating antiretroviral therapy (ART)-suppressed, HIV-infected humanized DRAGA mice following ART interruption. There were no adverse health outcomes associated with cell infusions. At 56 days post-treatment (DPT), 62.5% of CAR NK-treated and 50% of control NK-treated groups had viral loads below the detection limit compared with 0% of the saline control group. CAR NK-treated animals had, on average, 1.88 times higher peak NK cell levels than control NK-treated animals, and elevated levels of NK cells persisted up to 28 DPT in treated animals. Importantly, animals that had undetectable viral loads at 56 DPT had earlier viral rebound post-ART interruption that coincided with high levels of NK cells, suggesting that timing of treatment with viral recrudescence may play a role in efficacy. This is the first study evaluating NK cell therapies in hDRAGA mice and demonstrates the promise of NK cell therapies for curing HIV.

## Graphical abstract

**Figure:**
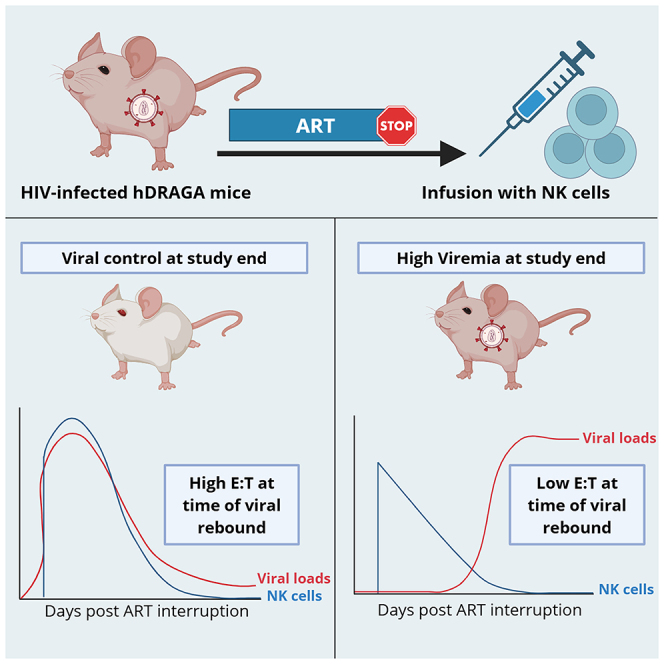

## Teaser

Skinner, Moriarity, and colleagues present a promising preclinical study of NK cell immunotherapies for treating HIV infections. NK cell treatment after antiretroviral therapy interruption led to HIV control in the majority of treated animals. A key factor associated with success was the coordination of treatment timing with the re-emergence of HIV.

## Introduction

Over 39.9 million individuals are living with HIV-1 worldwide.^1^ Although antiretroviral therapy (ART) has substantially reduced HIV-1-related morbidity, it is costly and requires lifelong adherence and non-adherence can lead to the emergence of ART-resistant HIV. Thus, innovative strategies to induce lifelong viral suppression are crucial. Chimeric antigen receptor (CAR) T cells have been effective in treating various cancers^2^^,^^3^^,^^4^^,^^5^ and are also being explored to treat HIV.^6^^,^^7^^,^^8^^,^^9^ A second-generation bispecific CAR has been developed containing the HIV-binding domain of CD4 linked to the carbohydrate recognition domain of mannose-binding lectin (MBL), both with high specificity for the HIV Envelope (Env) glycoprotein.^10^ The HIV-Env targeting CD4-MBL CAR shows high specificity and efficacy.^10^^,^^11^ We previously engineered therapeutic T cells to express a simian version of this CAR and the chemokine receptor CXCR5.^11^^,^^12^ Simian immunodeficiency virus (SIV) replication is concentrated within lymphoid B cell follicles,^13^^,^^14^^,^^15^^,^^16^^,^^17^^,^^18^^,^^19^^,^^20^ and yet most virus-specific cytotoxic cells within lymphatic tissues fail to express CXCR5,^20^ the necessary chemokine receptor for entry into these follicles. SIV-infected rhesus macaques were treated with CAR T cells that express CXCR5 and showed that these cells targeted SIV-producing cells and accumulated within lymphoid follicles. Treated animals also showed lower follicular viral RNA levels, and subsets of treated animals maintained lower viral loads compared with untreated control animals for the duration of the study.^11^^,^^21^ The findings suggest that CAR/CXCR5 T cells may lead to long-term viral suppression despite only persisting for 2–4 weeks, perhaps by allowing the host memory virus-specific T cell response time to accumulate at sufficient levels to suppress remaining viral replication.

As an alternative to T cells, natural killer (NK) cell therapies are attractive for many reasons.^22^ To name a few, peripheral blood (PB)-derived NK cells are easy to isolate and can be expanded to clinically relevant numbers using feeder cells expressing membrane-bound IL21 (mbIL21) and 4-1BBL (Clone 9 K562s).^23^^,^^24^^,^^25^^,^^26^^,^^27^ PB-NK cells do not express CD4, meaning they are not susceptible to HIV infection,^28^^,^^29^ and they do not induce graft-versus-host disease (GvHD), allowing them to be used as an allogeneic therapy.^30^^,^^31^^,^^32^ NK cells also naturally mediate the killing of virally infected cells,^33^^,^^34^^,^^35^^,^^36^^,^^37^^,^^38^^,^^39^^,^^40^^,^^41^ including HIV-infected cells,^42^^,^^43^^,^^44^ both through receptor-mediated cytotoxicity and antibody-dependent cell-mediated cytotoxicity.^45^^,^^46^ Moreover, NK cells have been evaluated for their use as an HIV treatment,^42^^,^^47^^,^^48^^,^^49^^,^^50^ including in human clinical studies in which a single infusion of haploidentical NK cells plus either IL-2 or N-803 (an IL-15 superagonist)^51^ was given to ART-suppressed people living with HIV (NCT03346499 and NCT03899480).^47^ The NK therapy was well tolerated and led to a decrease in the frequency of HIV vRNA+ cells in lymph nodes. This effect may be further enhanced by engineering NK cells to express HIV-specific CAR molecules to direct their targeting and enhance activation,^22^^,^^52^^,^^53^^,^^54^ as several CAR NK clinical trials have been conducted with promising results in cancers,^55^^,^^56^^,^^57^ and preliminarily for HIV.^58^^,^^59^^,^^60^ In African green monkeys, which naturally control SIV and remain asymptomatic, innate NK cells have been shown to accumulate in lymphoid follicles at high levels and are naturally CXCR5+.^61^^,^^62^ This finding supports the addition of CXCR5 to CAR NK cells for increased follicle-targeting capabilities.

There is a lack of animal models that recapitulate all components of HIV-1 infections in humans. SIV- or Simian-Human Immunodeficiency Virus (SHIV)-infected non-human primates are commonly used; however, their genetic diversity, particularly at immunogenetic loci, can determine their susceptibility to and maintenance of sustained infection.^13^^,^^14^^,^^63^^,^^64^^,^^65^^,^^66^^,^^67^^,^^68^ More recently, humanized immune system mouse models have been used to study HIV pathology, vaccines, and therapeutics.^69^ Humanized DRAGA (hDRAGA) mice develop functional human B and T cells and a limited number of NK cells, sustain HIV infections, demonstrate B cell immunoglobulin class-switching, and elicit specific human cellular and antibody responses after vaccination.^70^^,^^71^^,^^72^^,^^73^^,^^74^^,^^75^^,^^76^^,^^77^ Unlike other mouse models, hDRAGA mice develop secondary lymphoid tissues with follicle-like structures (FLSs) in the lymph nodes and spleen.^76^^,^^78^ Importantly, HIV virions were shown to replicate in follicular-like CD20^high^ areas of secondary lymphoid tissues of HIV-infected hDRAGA mice.^76^ We have recently demonstrated that HIV-infected hDRAGA mice are a valuable model for studying immune cell therapies.^78^

Given these findings, we engineered HIV-targeting CAR NK cells using non-viral DNA transposon technology and tested their safety and efficacy in infected hDRAGA mice. To further enhance the function of our CAR NK cell therapy, we armored our cells with soluble IL-15 and knocked out PD-1. Previous reports using innate immune effectors have demonstrated that IL-15 cytokine armoring can enhance *in vivo* persistence, function, and resilience.^47^^,^^79^^,^^80^ Additionally, our group and others have demonstrated that knockout (KO) of PD-1 may increase the cytokine production and cytotoxic function of NK cells, as well as improve their persistence.^52^^,^^57^^,^^80^^,^^81^ CAR/CXCR5/IL-15/PD-1^KO^ NK cells (for brevity, hereon referred to as CAR NK cells) were evaluated for functionality compared with feeder-cell-activated control NK cells *in vitro* and *in vivo*. We found that a subset of CAR- and control NK-treated animals had undetectable viral loads at study end. CAR NK-treated animals had, on average, 1.88 times higher peak NK cell levels than control-treated animals. Importantly, successful treatment was associated with earlier median viral rebound post-ART analytical treatment interruption (ATI), indicating the importance of treatment timing for NK cell therapies. This is the first study to evaluate NK cell therapies in hDRAGA mice for the treatment of HIV infection and demonstrates the promise of these therapies for curing HIV.

## Results

### NK cells were engineered to express HIV-specific CAR and CXCR5, secrete IL-15, and have PD-1 knocked out

**Figure 1:**
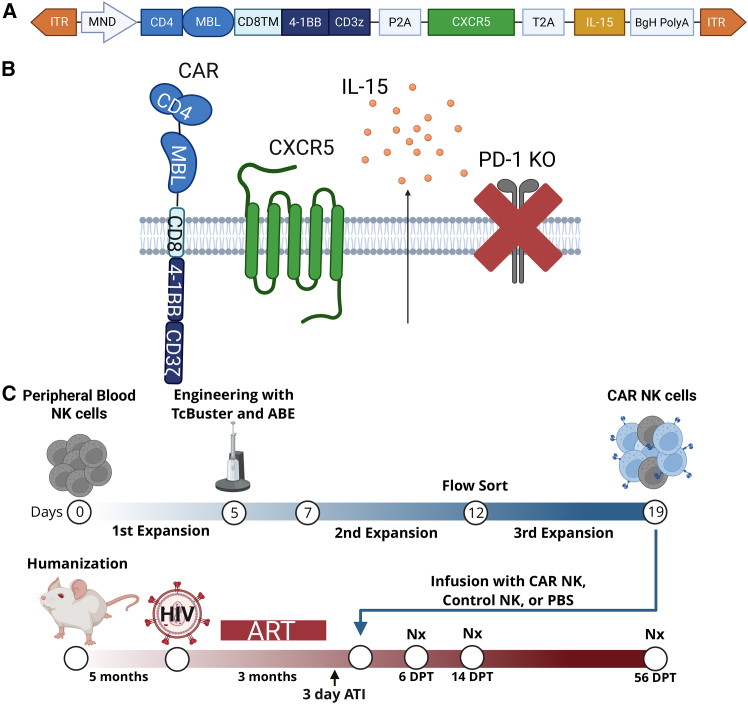
Overview of *in vitro* and *in vivo* study design (A) Schematic of transgene design: MND promoter, CAR (CD4-MBL-CD8TM-4-1BB-CD3ζ), CXCR5, IL-15, the BgH polyA tail, with separation mediated by P2A and T2A sites, and inverted terminal repeats (ITRs). (B) Schematic showing expression of all transgene components as well as base editor-mediated knockout of PD-1 on the CAR NK cell surface. (C) Schematic diagrams showing the overview of NK cell production and the overview of the mouse study timeline. Time points marked with Nx indicate planned necropsies.

CAR NK cells were engineered using the *TcBuster* non-viral DNA transposon system to express a polycistronic construct encoding CD4-MBL-CAR, CXCR5, and IL-15 with simultaneous PD-1 KO using an adenine base editor (ABE) (Figure 1). As a control, unedited NK cells were cultured in the same conditions but were not engineered. Following engineering and expansion, cells were sorted and then expanded for an additional week prior to functional testing (Figure 1).

**Figure 2:**
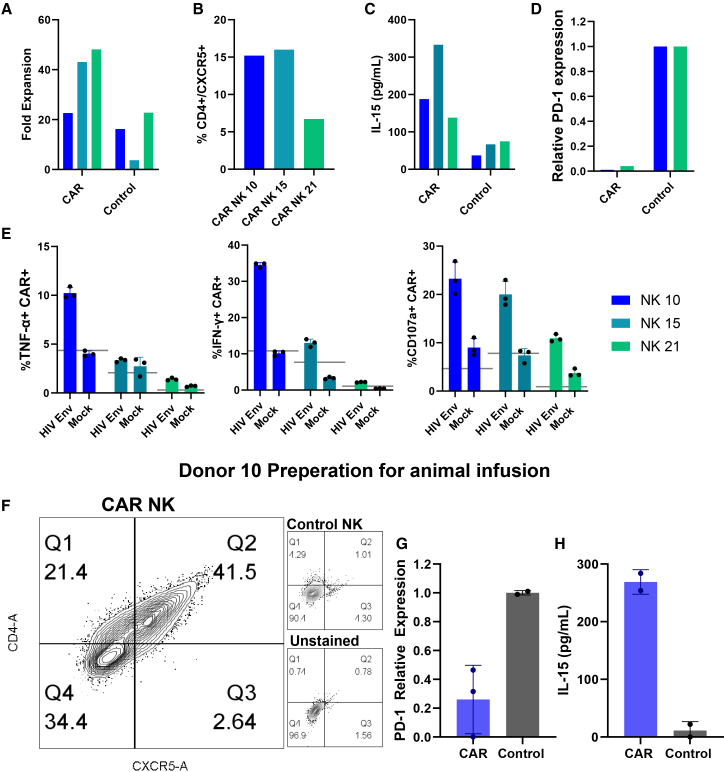
Engineered CAR NK cell products expressing CAR and CXCR5, with PD-1 KO and armored with IL-15, produced pro-inflammatory cytokines in response to antigen (A) Fold expansion of CAR and control NK cells over the third expansion for each donor (days 12–19 overall). (B) The percentage of NK cells from three donors that expressed CAR (CD4) and CXCR5 was detected by flow cytometry. Control NK cells showed less than 0.09% CAR+/CXCR5+. Cells were pre-gated sequentially on lymphocytes, singlets, live cells, CD56+, and CD3−. (C) IL-15 expression in CAR and control NK cells was detected through IL-15 ELISA of cell culture supernatant. (D) PD-1 knockout detected at the RNA level by reverse-transcription PCR (RT-PCR) with primers designed to detect the PD-1 sequence. (E) Pro-inflammatory cytokine (IFN-γ and TNF-α) and degranulation marker (CD107a) production measured in an intracellular cytokine staining (ICS) assay after CAR NK cells were cocultured with either HIV-Env or WT PBMCs. Bars represent medians with range. Gray lines represent the value of unstimulated NK cells with no targets present. CAR NK cells were also co-cultured with PMA/ionomycin as a positive control, and the values were 94.0%, 86.2%, and 78.8% for TNFɑ; 97.7%, 95.9%, and 98.8% for INFγ; and 90.9%, 84.4%, and 54.1% for CD107a for each donor, respectively. (F) Expression of CAR (CD4) and CXCR5 molecules in the product developed for infusion was detected using flow cytometry. The large box shows CAR NK cells, with smaller boxes on the right showing mock NK cells and unstained CAR NK cells, respectively. Cells were pre-gated sequentially on lymphocytes, singlets, live cells, CD56+, and CD3−. (G) PD-1 knockout detected at the RNA level by RT-PCR in CAR NK (blue) relative to control NK cells (black). (H) IL-15 expression in CAR NK (blue) and control NK (black) cells via IL-15 ELISA of cell culture supernatant.

Since there is variability between the functionality and expansion rates of NK cells from different donors,^82^ NK cells from three deidentified human donors (numbered here as donors 10, 15, and 21) were engineered and evaluated in functional assays (Figure 2). All three donor cells were successfully engineered to express the CAR and CXCR5. Donor 15 was determined to be unsuitable for production due to poor expansion of control NK cells (Figure 2A**)**, and as such we were unable to determine relative PD-1 expression from that donor. Despite this, subsets of CAR NK cells from all donors co-expressed CAR (detected by CD4) and CXCR5 (Figure 2B**)** and secreted IL-15 at levels above the control cells (Figure 2C**)** and donors 10 and 15 showed 96% and 99% KO of PD-1, respectively (Figure 2D**)**. Note that PD-1 protein loss results are not shown here, as PD-1 protein levels are minimally detected on the surface of NK cells from healthy individuals.^83^^,^^84^^,^^85^ Although PD-1 expression was expected to be low in the infusion cell preparation, we included PD-1 KO since there is elevated expression of PD-1 in HIV infection.^86^ CAR NK cells from each donor were also evaluated for their ability to produce pro-inflammatory cytokines (TNF-a and IFN-g) as well as the degranulation marker CD107a in response to HIV envelope-expressing peripheral blood mononuclear cells (PBMCs) or mock control PBMCs (Figure 2E**)**. In the presence of HIV-Env-expressing cells, donor 10 CAR NK cells showed the highest levels of TNF-α, IFN-ɣ, and CD107a production and were, therefore, selected for large-scale preparation of NK cells for infusion. CAR NK cells for the final preparation were prepared in the same manner for the three donors, except that they were sorted on CD4+ (part of CAR) and CD56+ after engineering to enrich the cell product. The resulting final CAR NK cell product was greater than 99% CD56+CD3− and had 41.5% dual expression of CAR and CXCR5 (Figure 2F**)** and a 74% (±23.7%) reduction in PD-1 expression compared with control NK cells (Figure 2G**)**. Cell culture supernatant of CAR NK cells also contained 268.818 (±10.97) pg/mL of IL-15, versus 11 pg/mL from control NK supernatant (Figure 2H**)**. These data demonstrate the successful engineering of CAR NK cells from the 3 donors and that CAR NK cells from some donors produce an elevated cytokine response to HIV-envelope-expressing target cells *in vitro*.

### Treatment with CAR or control NK cells was safe and associated with control of HIV viremia in a subset of treated animals

We evaluated the safety and efficacy of CAR/CXCR5, PD-1^KO^, IL-15-engineered (CAR) NK, and mock NK cell therapies in an *in vivo* study using HIV-infected hDRAGA mice (Figure 1A). Animals were sorted into groups taking into account their age, weight, time post-humanization, peak viral load, viral load area under the curve, and CD4 counts pre-infection (Figure S1). At the beginning of the study, 30 mice were included, with the intention of having 14 CAR-treated, 8 mock-treated, and 8 PBS-treated animals, with 6 animals in the CAR group intended for necropsy at 6 and 14 days post-treatment (DPT) to collect tissues for RNAscope and flow cytometry. Animal health and well-being were monitored for the duration of the study by visual inspection and body weight. Of the original 30 animals, six acquired severe adverse outcomes (Table S1) and required euthanasia prior to the intended study endpoint. Five of these animals were euthanized prior to being assigned treatment groups, and one additional mouse was euthanized after assignment in the PBS-treated group (Table S1). In all cases, observed symptoms were qualitatively consistent with chronic GvHD related to their engraftment with human stem cells (Table S1).^87^ Because of this loss of animals, we were forced to reduce the group sizes. We kept the CAR group at 14 animals to ensure we could still take animals for necropsy on days 6 and 14, and we left 7 animals in the PBS-treated group in order to ensure that statistics could still be run comparing treated animals with untreated animals. Unfortunately, this meant that we had to reduce the number of animals in the mock NK-treated group to 4, losing the ability to statistically compare this group with the others.

**Figure 3:**
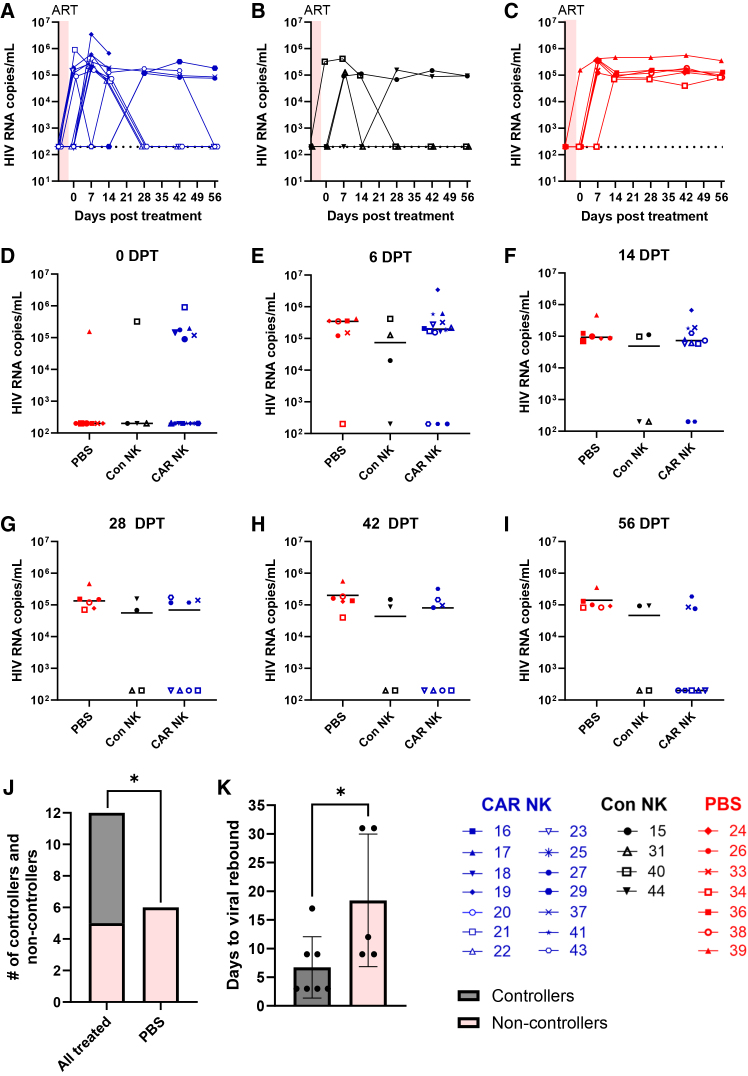
The majority of NK cell-treated animals had undetectable viral loads at 56 DPT Viral loads over time in (A) CAR NK-treated animals (blue), (B) control NK-treated animals (black), and (C) PBS-treated animals (red). Viral loads in CAR- (blue), control- (black), and PBS- (red) treated groups at (D) 0 DPT, (E) 6 DPT, (F) 14 DPT, (G) 28 DPT, (H) 42 DPT, and (I) 56 DPT. Lines represent median values. (J) Number of animals in either the treated (CAR and control NK) or untreated (PBS) groups at 56 DPT, with the number of controllers (gray) and non-controllers (pink) at 56 DPT (*p* = 0.0377), Fisher’s exact test of proportion within treated versus PBS groups. (K) Controllers (gray) and non-controllers (pink) at 56 DPT by number of days to viral rebound post-ART interruption (*p* = 0.0278, Mann-Whitney test). Mean values with SD are shown.

Viral loads were monitored throughout the study as the primary marker of therapeutic efficacy. HIV viral peak was observed in all mice by 2 weeks post-infection (Figures S2A–S2C). Mice were then given ART-containing chow from which they could free feed. Viral control was obtained in all mice between 4 and 8 weeks post-ART onset (Figures S2A–S2C). It was initially observed that mice were not eating the ART chow readily when it was placed in the hopper, and this led to a delay in viral control; however, adding the ART chow to paper cups on the bottom of the cage led to increased uptake and viral control in all animals. Once all mice were below the detection limit for HIV (less than 200 HIV RNA copies/mL of PB), they were infused intravenously with CAR NK cells or control NK cells at a dose of 1 × 10^5^–1.5 × 10^5^ cells/g or with saline buffer (PBS) solution. We found no signs of adverse symptoms related to treatment with CAR or mock NK cells compared with control animals. Three CAR NK-treated animals were taken to necropsy at days 6 and 14 each to collect tissue samples for RNAscope and flow cytometry, and, therefore, their viral loads end at the time of their necropsy (Figure 3A).

In all animals, viral loads rebounded to pre-ART levels between 3 and 28 days post-removal from ART (Figures 3A–3G; Figures S2A–S2C). At 28 DPT, 4 of 8 CAR NK-treated animals and 2 of 4 control NK-treated animals had undetectable viral loads, compared with 0 of 6 in the PBS control group (Fisher’s exact test, *p* = 0.1070) (Figure 3G**)**. Between 42 and 56 DPT, one additional animal in the CAR NK-treated group declined to undetectable viral loads (Figure 3I**)**. At study end, 62.5% (5/8) of CAR-treated, 50% (2/4) of control-treated, and 0% (0/6) PBS-treated animals had undetectable viral loads (Figures 3H and 3I**)**. For further analysis, we defined “controllers” as treated animals that had undetectable viral loads at study end (56 DPT) and “non-controllers” as treated animals that had positive viral loads at the same time point. The differences between the number of controllers and non-controllers between the CAR NK, control NK, and PBS group did not quite meet the criteria for statistical significance with the Fisher’s exact test (*p* = 0.0546). Importantly, however, there were undetectable viral loads in 58% (7/12) of NK-treated animals (CAR and control NK) compared with 0% (0/6) in the PBS group, demonstrating that overall, NK cell treatment led to a significant number of animals that controlled virus (*p* = 0.0377) (Figure 3J). Interestingly, in the animals that had undetectable viral loads by 56 DPT (controllers), the time to viral rebound following ART interruption was on average 15.5 days shorter compared with non-controllers (*p* = 0.0278) (Figure 3K**)**. On average, it took only 3.7 days for controllers to show detectable viremia following ATI, while it took 19.2 days for non-controllers. Thus, in all animals that controlled infection, viral rebound was earlier and closer to the time of therapeutic cell infusion. These data indicate that the NK cell treatments were only effective when the timing of cell infusion aligned with viral recrudescence.

### NK cell treatment does not significantly alter CD4+ T cell levels

**Figure 4:**
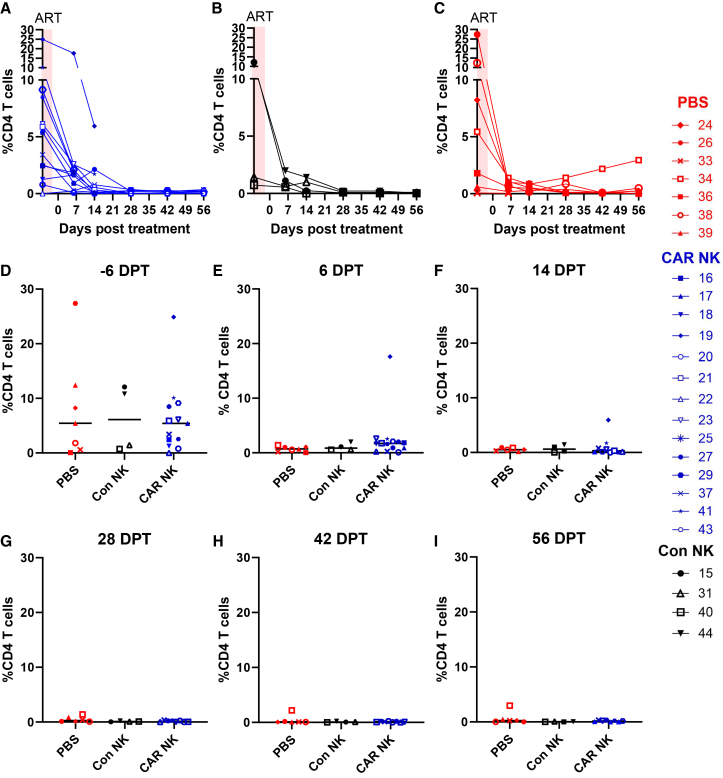
CD4 T cell levels declined in all groups following ATI Percentages of CD3+CD4+ cells out of lymphocytes over time in (A) CAR NK-treated animals (blue), (B) control NK-treated animals (black), and (C) PBS-treated animals (red). Percentages of CD4+ T cells out of lymphocytes in CAR- (blue), control NK- (black), and PBS- (red) treated groups at (D) −6 DPT, (E) 6 DPT, (F) 14 DPT, (G) 28 DPT, (H) 42 DPT, and (I) 56 DPT. Lines represent median values. CD4 percentages were determined by flow cytometry and pre-gated on lymphocytes, singlets, live cells, human CD45+, mouse CD45−, and CD3+ (see gating strategy in Figure S3).

CD4+ T cell levels were monitored as a secondary measure of treatment outcome, as decreasing CD4+ T cell levels are associated with HIV progression and CD4 maintenance is associated with therapeutic efficacy.^88^ One week after infection with HIV, 48% (12/25) of the animals showed an increase in CD4+ T cell levels; however, this effect was temporary, and by 4 weeks post-infection, 80% (20/25) of the animals showed a decrease in CD4 levels from baseline (Figures S2D–S2F). During ART, 48% (12/25) of the animals showed maintenance or an increase of CD4+ T cell levels, with the majority of the remaining animals showing a reduction in the rate of CD4 decline (Figures S2D–S2F). Following NK cell or PBS infusion and cessation of ART, all animals regardless of the treatment group saw a rapid reduction in CD4+ T cell levels, with all but one animal dropping below 3% of lymphocytes by 6 DPT (Figure 4). At 6 DPT, the median CD4+ T cell levels in the CAR group was 1.71%, compared with 0.86% in the control NK group and 0.72% in the PBS group; however, this difference was not significant (Figure 4E**)**. By 14 DPT, all three groups had median CD4+ T cell levels of 0.62% or lower (Figure 4F**)**. These data suggest that neither NK cell therapy made a lasting impact on CD4+ T cell levels.

### Peak NK cell concentration at the time of viral recrudescence is associated with viral control

**Figure 5:**
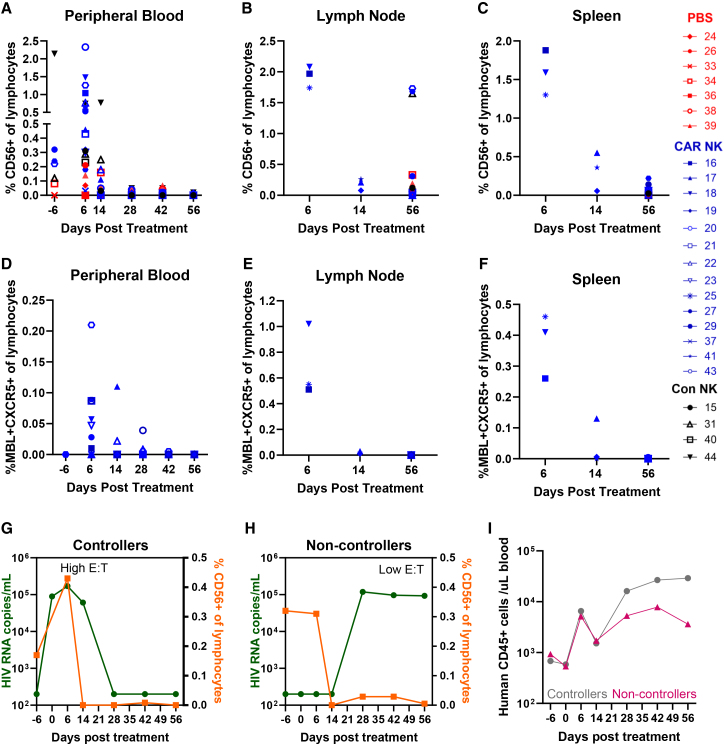
Peak NK cell concentration occurred at 6 DPT, with NK cells persisting up to 28 DPT in the peripheral blood NK (CD56+) cells in CAR- (blue), control- (black), or PBS- (red) treated groups from (A) peripheral blood, (B) lymph node (at necropsies), and (C) spleen (at necropsies). CAR+CXCR5+ NK cells over time in (D) peripheral blood, (E) lymph node (at necropsies), and (F) spleen (at necropsies). Flow was pre-gated on lymphocytes, singlets, live cells, human CD45+, mouse CD45−, CD3+, and then through CD56+ MBL+ CXCR5+ (see Figure S4 for gating strategy). Median viral loads (green) and NK cell levels (orange) post-treatment in (G) controllers and (H) non-controllers. (I) Median human hematopoietic cell counts (CD45+)/μL blood of controllers (gray) and non-controllers (pink).

Following infusion, hDRAGA mouse PB samples were regularly assessed for persistence of NK and CAR NK cells and spleen and lymph node samples were assessed at each necropsy time point (Figure 1). In the PB, NK cell levels peaked at 6 DPT for both the CAR- and control NK-treated groups, with the CAR NK-treated animals having 1.88 times higher concentration of NK cells than control NK-treated animals on average (Figure 5A). NK cells in treated animals were detected above untreated animal levels in some animals until 28 DPT (Figure 5A**)**. In addition to PB, spleen and lymph node samples were collected from 3 CAR-treated animals at 6 and 14 DPT, as well as from all remaining animals at 56 DPT (Figure 1). In these tissues, NK cell levels were slightly lower compared with the PB but similarly peaked at 6 DPT (Figure 5B**)**. In all but three animals, NK cells declined to background levels prior to 56 DPT (Figures 5B and 5C**)**. CAR/CXCR5+ NK cells were detected at low levels in the PB, with peak concentration occurring at 6 DPT (Figure 5D**)**. Low levels of CAR/CXCR5+ NK cells were detected in the spleen and lymph node at 6 DPT and declined to undetectable levels by 14 DPT in all but one animal’s lymph nodes (Figures 5E and 5F**)**. Spleen tissue sections from the three animals at 6 DPT were examined using RNAscope and immunohistochemistry to detect CAR+ cells, HIV vRNA+ cells, and CD20 (B cell marker used to delineate follicles). The three animals were found to have variations in their humanization and, therefore, in size and abundance of their FLSs. Animals with high levels of humanization showed more FLSs than animals with low levels of humanization. In sections with FLSs, the HIV vRNA+ cells were concentrated within the FLSs (Figure S4). In the sections examined from 6 DPT spleens, no CAR+ cells were detected above background levels (data not shown).

We noted that in NK cell-treated animals that controlled viral loads, the infused NK cell accumulation in the PB coincided with the time that HIV rebounded and peaked post-ATI (at 6 DPT). This resulted in relatively high median *in vivo* effector NK cell to target cell ratios (E:T) at viral peak (Figure 5G**)**. Conversely, in NK cell-treated animals that did not control HIV infection, the infused NK cells accumulated, peaked, and declined prior to HIV rebounding, resulting in relatively low median *in vivo* E:T at viral peak (Figure 5H**)**. We also noted that controllers had 1.39 times higher median peak NK cell levels compared with non-controllers; however, the observed difference was not statistically significant (Figures 5G and 5H). Taken together, these data suggest that NK cell levels at the time of viral rebound may play a role in viral control.

We also analyzed the human hematopoietic (CD45+) cell response after treatment in the responder and non-responder groups (Figure 5I**)**. All animals had increasing levels of human hematopoietic cells at 28 DPT, independent of the influx caused by treatment with NK cells, with this increase being somewhat more pronounced in the controller group. The hematopoietic cell levels remained high for the duration of the study in the controller group, while levels began to decrease in the non-controllers. At 56 DPT, human hematopoietic cells of the controllers were 8.07-fold higher than those of the non-controllers.

## Discussion

This is the first study evaluating NK cell therapies in hDRAGA mice. Our studies support a growing body of evidence^42^^,^^47^^,^^48^^,^^49^^,^^50^ indicating that NK cells show promise for the treatment of HIV infections. Our findings indicate the importance of timing the delivery of therapeutic cells with the timing of viral rebound. We found that both CAR NK cell and control NK cell treatments showed efficacy at suppressing HIV infections when infused at the time of viral recrudescence following ART interruption. In this study, 63% (5/8) of CAR NK-treated and 50% (2/4) control NK-treated animals had undetectable viral loads by 56 DPT compared with 0% (0/7) saline-treated animals. We did not find additional effects from the CAR-engineered cells above control; however, there was a statistically significant difference in the number of animals that controlled HIV between the NK treatment groups (CAR and control NK) and animals that received saline treatment (*p* = 0.0377). Interestingly, we also observed a statistically significant difference in the time to viral rebound between animals that controlled and did not control infection (*p* = 0.0278). In animals that controlled infection, median viral rebound occurred at the time the therapeutic cells were infused, resulting in relatively high *in vivo* E:T at viral peak. Conversely, in animals that did not control infection, in most cases, viral rebound occurred after the infused NK cells declined to very low levels, which resulted in relatively low *in vivo* E:T at viral peak. These findings suggest that NK treatment at the time of viral recrudescence may be important for the success of the treatment. These findings also support future studies in which additional doses of therapeutic cells are infused to ensure that relatively high E:T are achieved at the time of viral rebound in all animals.

Despite NK cell levels diminishing by 14–28 DPT, animals that controlled HIV had sustained undetectable viral loads until 56 DPT. HIV-specific T cell responses, particularly those of CD8 T cells, are believed to play an important part in spontaneous viral control^89^^,^^90^^,^^91^^,^^92^^,^^93^; however, they are often depleted during ART and are delayed in rebounding following ATI.^94^^,^^95^ CD8+ T cell responses have been observed in HIV-infected hDRAGA mice^76^; therefore, it is possible that NK therapies may have provided initial management of viral loads and allowed time for an hDRAGA adaptive immune response to occur. This is supported by the finding that human CD45+ cells proliferated in all treatment groups at 28 DPT. Once rebounded, the adaptive immune response may be better equipped to maintain control of HIV replication in animals with successful NK intervention, as controllers maintained higher hematopoietic cell levels for the duration of the study, while non-controller levels began to decrease. This theory also aligns with our previous studies, where we found sustained control of SIV in CAR/CXCR5 T cell-treated animals, long after the CAR T cells became undetectable.^21^ We cannot say for certain what specific cell types may have played a role in this mechanism, as we were limited by the volume of blood that we could safely collect from the animals in this study. Thus, further testing would be needed to verify this theory and determine which cells play a role in this sustained control. Nonetheless, these observations suggest that long-term control of HIV may be achieved with relatively short-acting cellular therapies.

Both CAR and control NK cells peaked at 6 DPT, with CAR NK cells having a 1.88 times higher peak concentration on average. Taken together, these findings suggest that CAR NK cells were more proliferative and persisted longer *in vivo* compared with the control NK cells, which may have been due to the expression of IL-15 by CAR NK cells.^47^^,^^79^^,^^80^ Further studies are warranted to examine the impacts of IL-15 on the proliferation and persistence of CAR NK therapies for HIV or using IL-15 super-agonists like N803 or other fused membrane IL-15 receptors to further enhance this effect.^96^^,^^97^

Additional doses of NK cells could lead to improved viral suppression in the animals that did not control HIV. Since timing NK cell treatment with viral rebound seems to be important for viral control, infusing a second dose of NK cells could help improve the odds of treating at the time of viral rebound in all animals, including those with delayed viral rebounds. This theory aligns with NK cell therapies to treat myeloma, where multi-dosing regimens were shown to improve NK treatment efficacy.^98^^,^^99^^,^^100^ In addition to multiple doses of NK cells, an earlier dose may lead to viral suppression without viral rebound occurring. Indeed, Kim et al. found that treating HIV-infected mice with allogeneic PB-derived NK cells 1 and 6 days after ART interruption led to a significant delay in viral rebound and significantly lower numbers of rebound barcode HIV variants.^48^

We found no evidence of adverse outcomes related to NK cell treatment. Like most humanized mouse models, hDRAGA mice risk developing chronic GvHD.^101^ It is of interest that the 6 animals that had GvHD-like symptoms were all female and were humanized with male donor cells. Although it is widely considered safe to transplant female mice with male stem cells,^102^^,^^103^ some studies in human stem cell transplant have indicated that sex-mismatched stem cell engraftments can lead to increased risk of developing GvHD.^104^^,^^105^ Additionally, 5 of the 6 mice were humanized with cells from the same human stem cell donor, and donor-specific factors have been known to increase the risk of developing GvHD.^106^ It is regrettable that the development of GvHD prevented us from including the group sizes originally intended for this study. However, hDRAGA mice are still one of the most robust models for studying therapies for HIV at this time. Thus, future studies of immune cell therapies using this model should be aware of the impacts of GvHD and anticipate approximately 20% animal loss over a 5-month study duration.

We detected low levels of CAR NK cells in disaggregated spleen and lymph nodes from treated animals by flow cytometry, with the highest levels detectable at 6 DPT. However, no CAR+ cells were detected by RNAscope in spleen sections that were examined. Taken together, these data indicate that while the therapies showed efficacy in this model system, they failed to accumulate at high levels in secondary lymphoid organs despite expressing CXCR5. We have previously evaluated the functionality of CXCR5 on CAR/CXCR5 T cells and showed that these cells migrate and accumulate in lymphoid follicles *in vivo*.^11^^,^^21^ We suspect that NK cells may not have accumulated well in lymphoid tissues due to a lack of other necessary chemokine receptors for lymphatic tissue entry. NK cell subsets express CXCR1, CXC3R1, and ChemR23, and, at low levels, CCR7, allowing them to migrate to lymphatic tissues in inflammatory conditions,^107^ like HIV infection.^108^^,^^109^ To further increase the levels of NK cells migrating to these lymphatic tissues, in future studies, we might increase the expression of CCR7 and L-selectin on PB-NK cells, which may lead to increased migration to lymphatic tissues. Transient expression of CCR7 combined with CXCR5 may be ideal for B cell follicle homing, as other cell types like T follicular helper cells use CCR7 to enter lymphatic tissues and then downregulate CCR7 and rely on CXCR5 to enter follicles.^110^^,^^111^^,^^112^ Somanchi et al. have previously shown that NK cells can be made to transiently express CCR7 via trogocytosis following co-culture with CCR7-overexpressing K562 feeder cells.^113^ Using this method, CCR7 expression returned to baseline levels after 72 h. Alternatively, mRNA transfection could be used to express CCR7, L-selectin, and CXCR5, which have been shown to improve homing of NK cells to the lymph nodes.^114^ Future studies with NK cells engineered with CCR7 and CXCR5 are warranted to determine whether these homing molecules are sufficient to clear the HIV reservoirs in lymphatic tissues.

Additional strategies to achieve better long-term control of infection include CAR NK or NK cell immunotherapy, combined with other strategies aimed at eliminating the HIV/SIV reservoir. Such therapies might include latency reversal agents to reactivate latent HIV-infected cells,^48^^,^^115^^,^^116^^,^^117^ Bi- or tri-specific Killer cell Engagers (BiKEs/TriKEs) to connect immune cells with HIV-expressing cells,^118^^,^^119^^,^^120^ or agents that modify the immune system, such as an IL-15 super-agonist^47^^,^^96^ or rapamycin.^121^^,^^122^

Taken together, these studies demonstrate the promise of NK cell therapies for treating HIV. We showed that CAR and control NK cells are safe and were associated with undetectable viral loads in the majority of treated animals. Importantly, we observed that timing of treatment with viral recrudescence was associated with viral control. Future studies could explore multiple rounds of NK cell infusions to achieve superior efficacy. With refinement of treatment timing, immunotherapeutic NK cells could lead to long-term suppression of HIV without the use of lifelong ART.

## Materials and methods

### Production of engineering reagents

ABE8e plasmid was obtained from Addgene (https://www.addgene.org/138489/) and cloned into a pmRNA vector. ABE8e mRNA was produced by TriLink BioTechnologies. Nanoplasmid encoding the bispecific CD4-MBL CAR with the 4-1BB intracellular domain, CXCR5, and IL-15 was synthesized by Aldevron. Specific sequences are included in Figure S5.

### Donor NK cell isolation and expansion

PBMCs from three de-identified healthy human donors (labeled as donors 10, 15, and 21 for the purpose of distinguishing) were obtained via automated leukapheresis (Memorial Blood Centers, Minneapolis, MN, USA) and further isolated using a Ficoll-Hypaque (Lonza) gradient. NK (CD56+/CD3−) cells were isolated using CliniMACS, following the manufacturer’s instructions. NK cells were cultured in CTS AIM V SFM (Thermo Fisher) supplemented with 5% CTS Immune Cell SR (Gibco), penicillin/streptomycin (P/S), and IL-2 (100 IU/mL), as previously described.^52^ Activation of NK cells was achieved by co-culturing NK cells with X-irradiated (100 Gy) feeder cells (K562 cells expressing membrane-bound IL-21 and 4-1BBL) at a 1:2 ratio of NK cells to feeders. Cells were supplemented with additional medium and IL-2 up to 100 IU/mL every 2–3 days.

### Production of CAR/CXCR5/PD-1KO/IL-15 NK cells

CAR NK cells were engineered using the *TcBuster* non-viral DNA transposon system to express a polycistronic construct encoding CAR, CXCR5, and IL-15. An ABE and a guide RNA complementary to the PD-1 gene (CACCTACCTAAGAACCATCC) were utilized to knockout PD-1 expression. Isolated NK cells were expanded for 5 days following the culture and activation protocol above. Cells were electroporated using the MaxCyte (Thermo Fisher) with transposase mRNA, nanoplasmid DNA, ABE8E mRNA, and guide RNA as previously described^55^ or electroporated with no additional reagents for control NK cells. After a 2-day recovery, cells were expanded for an additional 7 days following the procedure described above. In the final preparation of CAR NK cells for infusion, cell sorting was used after the 2-day recovery, and cells were sorted on CD56+CD4+ using the MACSQuant Tyto Cell Sorter (Miltenyi Biotec).

### Cell culture of target cells

Inducible truncated HIV-Env P815s: the P815 truncated HIV Env cell line was produced by retronectin-mediated transduction of P815 cells (a gift from Dr. Geoffery Hart) using a lentivirus produced from a tet-inducible truncated HIV Envelope plasmid (kindly provided by Dr. Alon Herschhorn; Tet-One Inducible Expression System, Clontech) and psPax2 and VSV-G plasmids. Puromycin-resistant cells were cloned by limiting dilution to produce a stable cell line. Cells were cultured in DMEM + 10% tet-free FBS + 1% P/S and maintained at a density of 0.7e5-1e6 cells/mL medium. Selective pressure for cells containing the inducible HIV-Env construct was maintained by adding 1 μg/mL puromycin to the culture. HIV Env expression was induced by adding 1 μg/mL of doxycycline to the culture for 72 h. Expression of HIV Envelope was assessed by flow cytometry.

Trunc-HIV Env expressing PBMCs: the HIV Envelope gene, truncated 145 amino acids at the C terminus, was inserted into the pMSGV gammaretroviral plasmid by Gene Universal. Pseudotyped gammaretrovirus containing truncated HIV-Env was produced by Lipofectamine-mediated transfection of 293T cells cotransfected with pBS-CMV-gagpol, RD114, and pMD.g, as described previously.^11^ PBMCs from a human donor were transduced with HIV-Env pseudotyped gammaretrovirus as previously described.^11^^,^^123^ As an assay control, NK cells were subjected to the same culture conditions with no virus present. HIV-Env PBMCs were evaluated for expression and frozen for future expansion and use. Prior to running assays, cells were thawed and expanded in a GREX with 50 IU IL-2 + 55 μM beta-mercaptoethanol (Gibco) added to the medium for an additional 4 days. Expression of HIV Envelope was assessed on a CytoFLEX flow cytometer after gating on alive, single cells.

### Pro-inflammatory cytokine and degranulation marker assay

Antigen-specific production of TNFα, IFNγ, and CD107a was assessed by coculturing CAR NK cells (effector) with either Trunc-HIV-Env PBMCs or mock PBMCs at E:T of 1:1. As a positive control, some CAR NK cells were cultured with PMA (50 ng/mL) and ionomycin (1 μg/mL) for maximum release. Anti-human CD107a (BioLegend, H4A3, BV421) was added and cells were incubated for 1 h at 37°C, at which point brefeldin A (BioLegend) and monensin (BioLegend) were added, and the cells were incubated for 3 h at 37°C. Intracellular cytokine staining was performed for TNF-α (BioLegend, MAb11, BV650) and IFNγ (BioLegend, B27, PerCP/Cyanine 5.5) prior to surface staining for MBL (Invitrogen, 3E7), CXCR5 (Invitrogen, MU5UBEE, PE), and CD56 (Immunotech, N901, PC5.5). Samples were acquired on a CytoFLEX (Beckman) flow cytometer and analyzed via FlowJo v.10.2 software (Becton Dickinson). Cells were gated on lymphocytes, single cells, CD56+ MBL+ CXCR5+, and then TNF-α+, IFN-γ+, or CD107a+.

### Analysis of PD-1 expression

Expanded CAR and control NK cells were harvested, and total RNA was isolated using the RNeasy Mini Kit (QIAGEN, 74104) following the manufacturer’s instructions. 1 μg of RNA was reverse-transcribed into cDNA with the SuperScript IV RT Kit (Life Technologies, 18090050). qPCR reactions were performed using SsoAdvanced Universal SYBR Green Supermix (Bio-Rad, 1725274) with the following primers: PD-1 forward (CCCTGGTGGTTGGTGTCGT) and reverse (GGCTCCTATTGTCCCTCGTGC); β-actin forward (AGGCACCAGGGCGTGAT) and reverse (TGGGGTACTTCAGGGTGAGGA). Amplification was carried out on a CFX96 Real-Time PCR System (Bio-Rad), and data were analyzed using CFX Manager 3.1 software (Bio-Rad, CA, USA). Relative gene expression was calculated using the comparative Ct (ΔΔCt) method with β-actin as the internal control, and the percentage reduction in expression was calculated as %Reduction = (1 − Relative Expression) × 100.

### Detection of IL-15 production

CAR and control NK cells were collected following their third expansion. Cells were centrifuged, and the supernatant was collected. Human IL-15 ELISA kits (Abcam) were used to determine IL-15 concentration in the supernatant following the manufacturer’s instructions.

### HIV infection and treatment of DRAGA mice

DRAGA mice (HLA-A2. HLA-DR4. RAG1 KO. IL-2R g c KO. NOD) are on an NRG background and have transgenes for HLA-A2 and HLA-DR4, allowing for enhanced donor-specific engraftment with CD34+ human hematopoietic stem cells from cord blood.^70^ They were bred and humanized at the Veterinary Service Program at WRAIR/NMRC before being transported to the University of Minnesota. Thirty humanized DRAGA (hDRAGA) mice (18 male and 12 female), humanized from two human donors, were housed under specific pathogen-free conditions. Following a 1-week quarantine period, hDRAGA mice were evaluated for the reconstitution levels of human immune cells and then infected intraperitoneally with 20,000 tissue culture infectious dose 50 (TCID50) of HIV-1Ba-L in 200 μL PBS, as previously described.^124^ Four weeks after infection, the mice were switched from standard feed to 1/2″ pellets of irradiated Teklad chow 2020X containing 1,500 mg emtricitabine, 1,560 mg tenofovir disoproxil fumarate, and 600 mg raltegravir per kg (Research Diets, New Brunswick, NJ), as described by Tsai et al.^125^ in a hopper. It was initially observed that mice were not eating the ART chow readily when it was placed in the hopper, so the ART chow was instead provided in paper cups on the ground. Once all mice had undetectable HIV viral loads, they were switched back to standard Picolab dry feed. Animals were sorted into infusion groups taking into account their age, weight, time since infusion, peak viral load, viral load area under the curve, and CD4 counts pre-infection (Figure S1). Three days post-ART analytical treatment interruption (ATI), the mice received an intravenous administration of CAR NK cells, control NK cells, or PBS at a dose of 1 × 10^5^−1.5 × 10^5^ cells/g. Animals were euthanized at 6, 14, and 56 DPT via carbon dioxide inhalation followed by cervical dislocation. Mice were visually inspected daily for clinical scores and weighed weekly to monitor health and well-being.

### Blood and tissue collections from hDRAGA mice

Blood samples (up to 150 μL) were collected from the facial veins with the following frequencies: weekly for the first four weeks post-infection, and then biweekly from the start of ART until day 56 post-treatment. The blood samples were separately processed for isolating PBMCs and plasma, as previously described.^78^ Spleen and lymph nodes were collected from animals at the time of necropsy (6, 14, and 56 DPT) and divided into two parts. One part was disaggregated and lysed to remove red blood cells before being processed as described below for flow cytometric analysis. The remaining spleen and lymph nodes were fixed in 4% paraformaldehyde and embedded in paraffin blocks by the U of M Clinical and Translational Science Institute and sectioned at 5 μm as previously described.^21^

### Whole blood cell counts and phenotype analysis

Cell counts were performed using 50 μL of whole blood, as described previously.^78^ Briefly, red blood cells were lysed using ACK lysing buffer (Gibco), and samples were washed. The cells were resuspended in 150 μL of PBS, and 50 μL of this suspension was mixed with a known concentration of AccuCheck counting beads (Invitrogen). The remaining resuspended cells were stained for flow cytometry as described below.

### Flow cytometry of hDRAGA samples

Samples collected from spleen and lymph nodes were disaggregated and lysed. Samples from PB, spleen, and lymph nodes were then characterized using antibodies to stain for the following epitopes: CD4 (BD Biosciences, M-T477, FITC), CXCR5 (Invitrogen, MU5UBEE, PE), MBL (Invitrogen, 3E7) conjugated to Alexa Fluor 647, CD3 (BD Biosciences, SP34.2, AF-700), CD56 (Immunotech, N901, PC5.5), and CD8 (Invitrogen, 3B5, PacBlue). Anti-mouse CD45 (BioLegend, 30-F11, BV785) and anti-human CD45RA (BD Biosciences, 5H9, PE-Cy7) were used to distinguish mouse and human hematopoietic cells. Samples were acquired on a Beckman Coulter CytoFLEX cytometer, and a minimum of 10,000 events were collected for each sample. All data were analyzed using FlowJo software v. 10.2 (Becton Dickinson).

### Plasma HIV-1 viral load determination

Plasma viral loads were detected as previously described.^78^ Up to 50 μL of plasma was used for RNA extraction using a QIAamp Viral RNA Mini Kit (QIAGEN) according to the manufacturer’s instructions. HIV-1 vRNA quantitation was carried out using real-time PCR with an HIV Quantitative TaqMan RT-PCR Detection Kit (Norgen Biotek Corp.) on a CFX96 Real-time PCR System (Bio-Rad). Data collection and analysis were performed using CFX Manager 3.1 software (Bio-Rad, CA, USA), as previously described.^78^

### RNAscope and immunohistochemistry

RNAscope *in situ* hybridization (ACD) and immunofluorescence were performed following manufacturer specifications and as previously described.^21^ Briefly, 5-μm tissue sections were deparaffinized, pre-treated, washed, dehydrated in absolute ethanol, and air-dried. Sections were incubated with pre-warmed premixed target probes targeting HIV-Env and the CD4-MBL region of the CAR molecule and amplified. Opal 570 and Opal 650 were used for HIV-Env and CD4-MBL, respectively. For immunofluorescence staining, sections were washed, blocked, and incubated with primary rabbit anti-human CD20 antibody (Abcam, EP459Y) at 1.12 μg/mL. Sections were washed and incubated with 10 μg/mL Alexa 488-labeled goat anti-rabbit secondary antibody (Invitrogen) diluted in a blocking buffer for 1 h at room temperature. After washing, sections were counterstained with 1 μg/mL DAPI and mounted in Prolong Gold (Invitrogen).

### Quantitative image analysis for RNAScope

Sections were imaged using a Nikon Ti-E confocal microscope. FLSs and extrafollicular areas were delineated by morphology, with FLSs identified as clusters of closely aggregated CD20+ cells. QuPath software was used to perform cell counts, delineate tissue areas, and define CAR+DAPI+ and HIV+DAPI+ cells above background expression levels on negative-stained tissues. For these analyses, for each animal, 3 tissue sections were examined, and all FLSs (if any existed) were examined in each tissue section.

### Statistical analysis

All statistical analyses were performed using GraphPad Prism v.10. Mann-Whitney or Fisher’s exact tests were used where appropriate. *p* < 0.5 was considered significant. Standard deviations or ranges are indicated by the ± symbol and specified in the text where appropriate.

## Data availability

The data underlying this article will be shared on reasonable request to the corresponding author.

## Acknowledgments

This work was supported by amfAR Target (110411) grant to P.J.S., V.V., and B.S.M.; 10.13039/100000060NIAID R01 (AI161017) to P.J.S. and B.S.M.; and the Military Infectious Diseases Research Program (MI230024 and MI240008) grants to S.A.C. S.A.C. is a US Federal employee. The work of this individual was prepared as part of official government duties. Title 17 U.S. C. §105 provides that “copyright protection under title is not available for any work of the United States Government.” Title 17 U.S.C. §101 defines US Government work as work prepared by a military service member or employee of the US Government as part of that person’s official duties. The views expressed are those of the authors and do not necessarily reflect the official policy or position of the 10.13039/100009896Department of the Navy, Department of the Army, Department of War, or the US Government. All animal procedures reported herein were conducted under IACUC protocols approved by the 10.13039/100010203Walter Reed Army Institute of Research/10.13039/100007680Naval Medical Research Command (WRAIR/NMRC) and the UMN (protocol ID:2401-41649A) in compliance with the Animal Welfare Act and by the principles outlined in the “Guide for the Care and Use of Laboratory Animals,” Institute of Laboratory Animals Resources, 10.13039/100013101National Research Council, National Academy Press, 2011. This material has been reviewed by the 10.13039/100007680Naval Medical Research Command. The following reagent was obtained through the 10.13039/100000002NIH HIV Reagent Program, Division of AIDS, NIAID, NIH: human immunodeficiency virus type 1 HIV-1_Ba-L_ virus (ARP-510), contributed by Dr. Suzanne Gartner, Dr. Mikulas Popovic, and Dr. Robert Gallo. Anti-CD3 (OKT3) used in the current study was provided by the 10.13039/100000054National Cancer Institute’s Biological Resources Branch Preclinical Repository. Some figures were created with BioRender. We thank Joshua Krueger for the preparation of plasmid reagents, Isabelle Finholm for her assistance with RNAscope, Dr. Geoffrey Hart for the kind gift of the P815 cells, and Dr. Alon Herschorn for the gift of the tet-inducible HIV-Envelope plasmid. B.S.M. also acknowledges funding from Office of Discovery and Translation, 10.13039/100000002NIH grants (R01AI146009, R01AI161017, P01CA254849, P50CA136393, U24OD026641, U54CA232561, P30CA077598, and U54CA268069), 10.13039/100000005DOD grants (HT9425-24-1-1005, HT9425-24-1-1002, and HT9425-24-1-0231), and the 10.13039/100000885Children's Cancer Research Fund, the Fanconi Anemia Research Fund, and the Randy Shaver Cancer and Community Fund. There is no objection to its presentation and/or publication.

## Author contributions

L.K.T. and P.P.-o. were responsible for data curation, investigation, validation, and visualization. L.K.T. was the lead in analyzing data with P.P.-o. in a supporting role. J.-W.C. was involved in data curation, investigation, methodology, resources, and validation. M.E.C., I.G.-B., K.L.B., Z.E.Q., and A.F.K. all contributed in a supporting role to data curation, investigation, and validation. A.K.R. contributed to formal analysis and validation. M.S.P. was responsible for conceptualization, project administration, methodology, and resources. S.A.C. was responsible for conceptualization, methodology, funding acquisition, and resources. B.S.M., P.J.S., and V.V. were responsible for conceptualization, funding acquisition, resources, supervision, and project administration. L.K.T. was the lead in writing the manuscript, and all authors were responsible for reviewing and editing of the manuscript.

## Declaration of interests

P.J.S. is the co-founder of and has equity in MarPam Pharma LLC.

## Supplemental information

Document S1. Figures S1–S5 and Table S1

Document S2. Article plus supplemental information
