# Supplementary material for: NK cell immunotherapy after analytic treatment interruption is associated with HIV viral control
Source: Mol Ther Adv. 2026 Jun 11;34(3):201778. doi: 10.1016/j.omta.2026.201778 (PMC13343159; doi:10.1016/j.omta.2026.201778)
Supplement: Document S1. Figures S1–S5 and Table S1 [file mmc1.pdf]

OMTA, Volume *34*

Supplemental information

NK cell immunotherapy after analytic treatment interruption is associated with HIV viral control

Liliana K. Thron, Pongthorn Pumtang-on, Jae-Woong Chang, Maxwell E. Cantor, Ian Gorrell-Brown, Kelsie L. Becklin, Zoe E. Quinn, Ahmad F. Karim, Aaron K. Rendahl, Mary S. Pampusch, Sofia A. Casares, Vaiva Vezys, Branden S. Moriarity, and Pamela J. Skinner

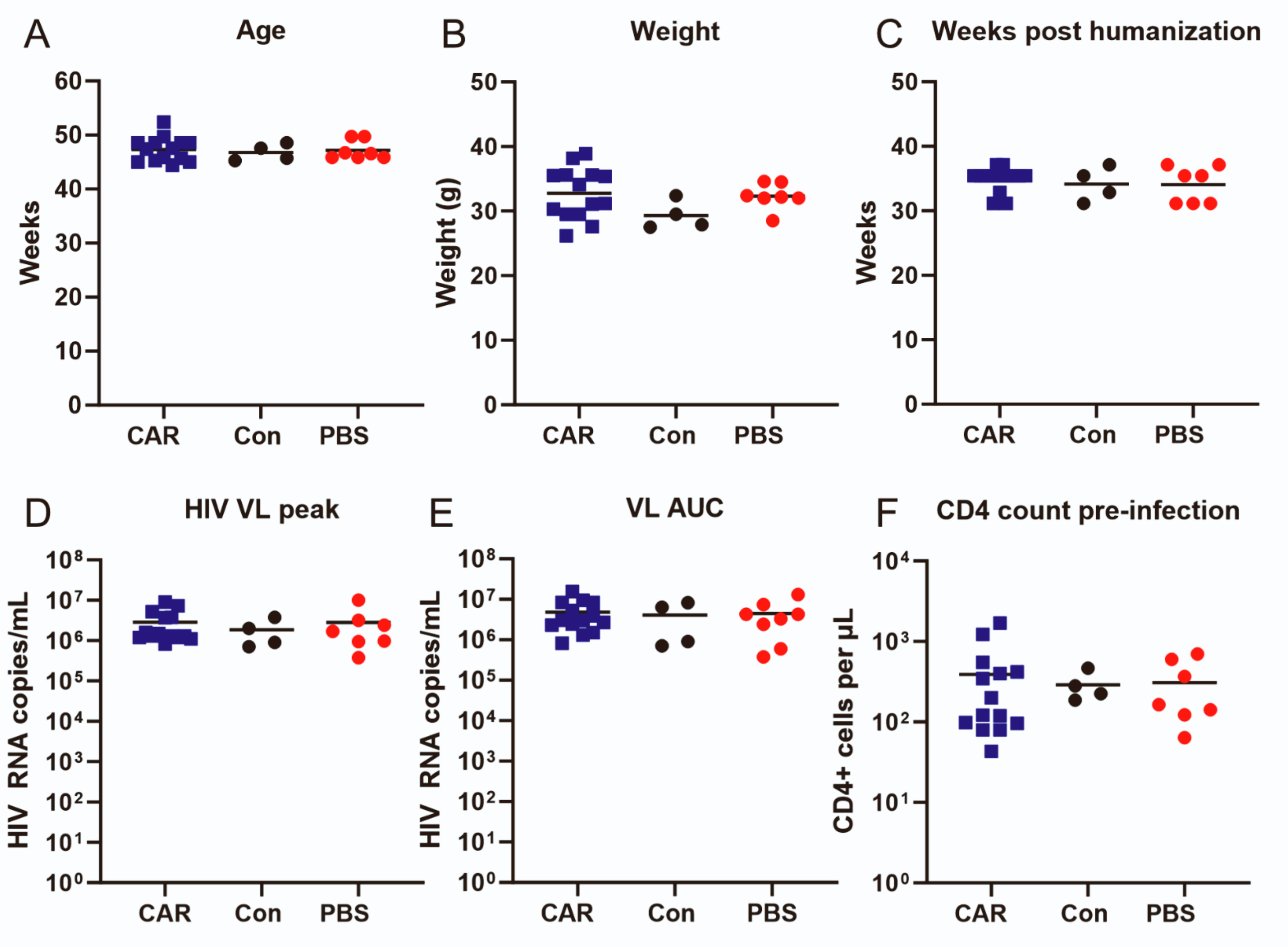

Figure S1: DRAGA animal grouping allocations. DRAGA mice were grouped for either CAR (blue), control (black), or PBS (red) treatment based on A) age at the time of treatment, B) weight at the time of treatment, C) amount of time since humanization D) HIV vRNA peak prior to ART treatment, E) Area under the curve of viral loads prior to ART treatment, and F) CD4 count prior to infection.

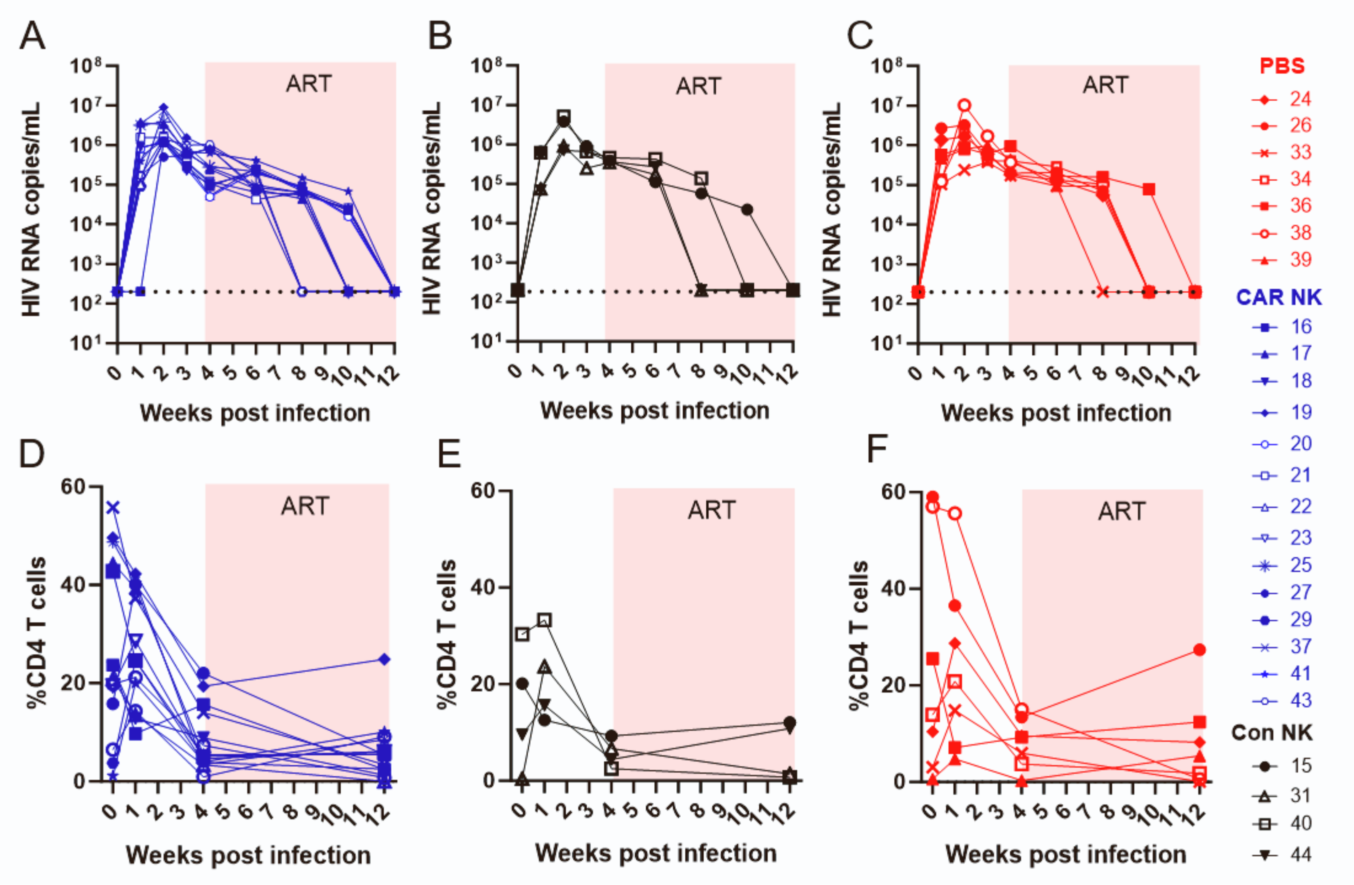

Figure S2: Viral loads and CD4 T cell levels before infusion of NK cell therapies. Viral loads over time in A) CAR NK-treated animals (blue), B) control NK-treated animals (black), and C) PBS-treated animals (red). % CD4 T cells out of lymphocytes over time in D) CAR NK-treated animals (blue), E) control NK-treated animals (black), and F) PBS-treated animals (red).

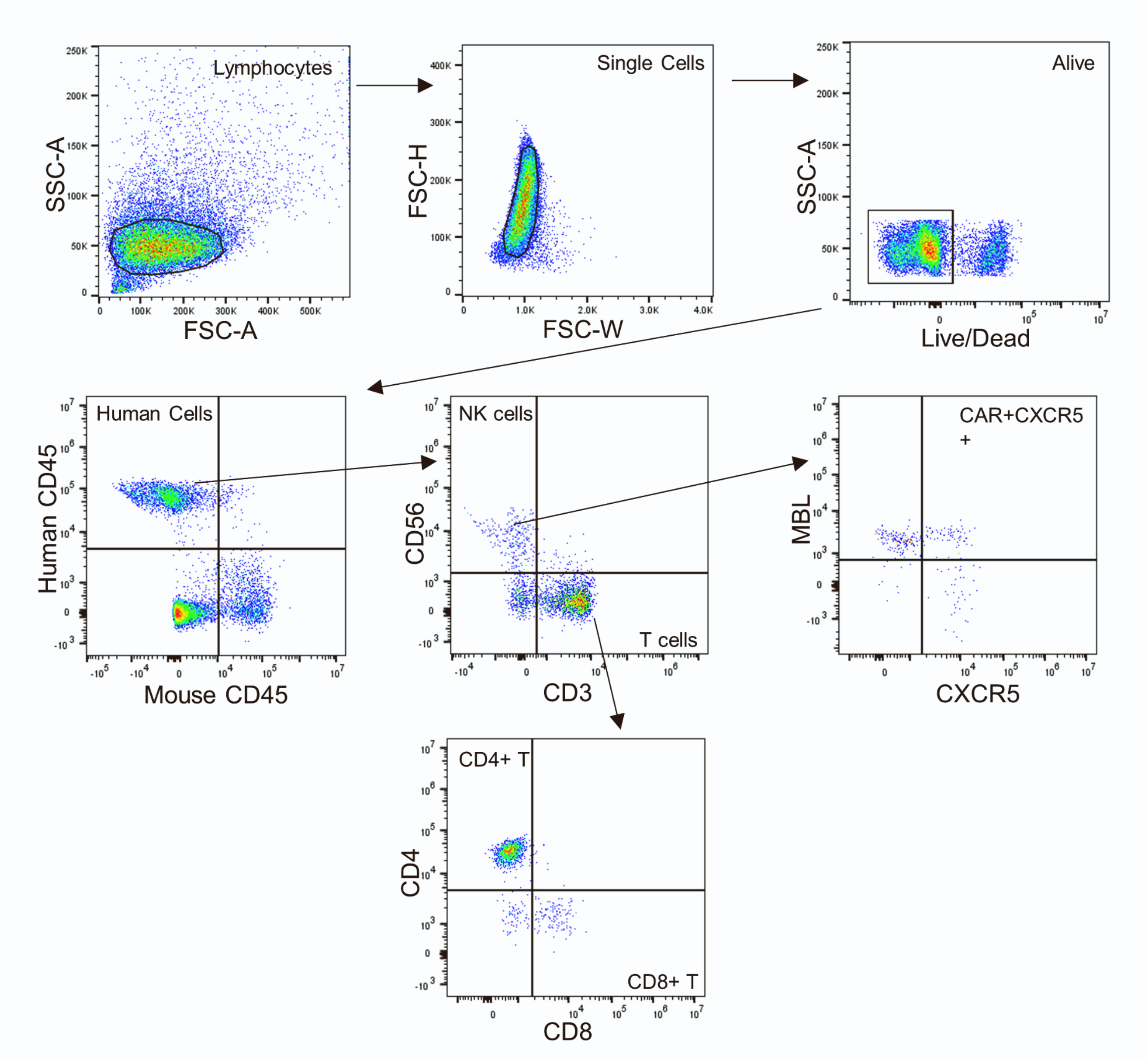

Figure S3: Flow cytometry gating strategy for DRAGA mouse study. Cells were gated Lymphocytes, singlets, alive cells, Human CD45+, Mouse CD45-. NK cells were gated CD56+, CD3-, and CAR NK cells were further identified by gating MBL+ CXCR5+. T cells were identified by gating CD3+ CD56-, and then CD4 or CD8+.

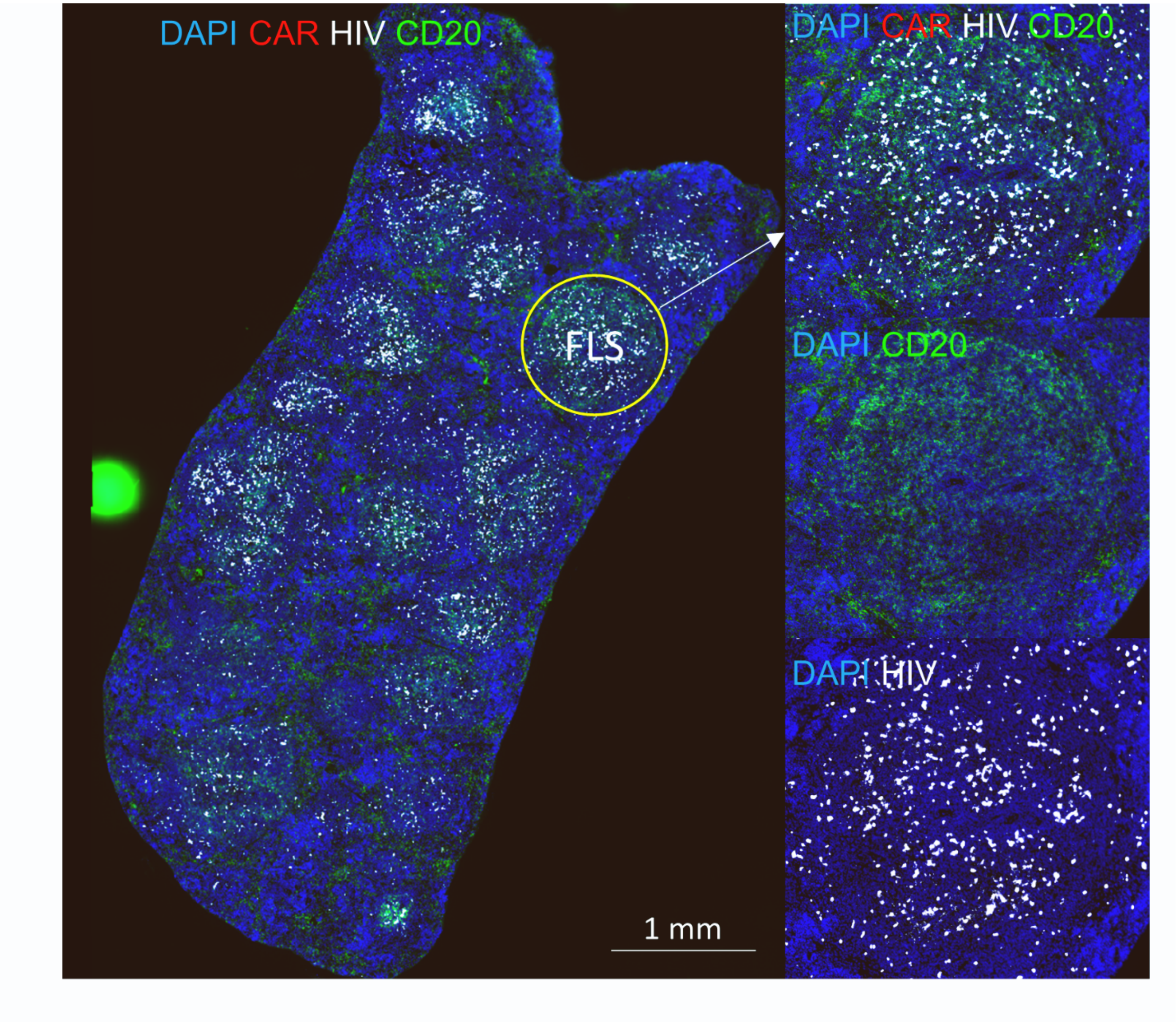

Figure S4: HIV vRNA+ cells were concentrated in the FLS regions of the spleen at 6 DPT. A) Spleen from DRAGA 25 at 6 DPT (necropsy) stained for DAPI (blue), CAR+ (red), HIV+ (white), and CD20+ (green). FLS were identified by CD20+ morphology. On the right, enhanced images of the follicle with staining as indicated.

MVRGVPFRHLLLVLQLALLPAATQGKKVVLGKKGDTVELTCTASQKKSIQFHWKNSNQIKILGNQGSF

LTKGPSKLNDRADSRRSLWDQGNFPLIIKNLKIEDSDTYICEVEDQKEEVQLLVFGLTANSDTHLLQGQS

LTLTLESPPGSSPSVQCRSPRGKNIQGGKTLSVSQLELQDSGTWTCTVLQNQKKVEFKIDIVVLAFQKAS

GGGGSKQVGNKFFLTNGEIMTFEKVKALCVKFQASVATPRNAAENGAIQNLIKEEAFLGITDEKTEGQF

VDLTGNRLTYTNWNEGEPNNAGSDEDCVLLLKNGQWNDVPCSTSHLAVCEFPIAAATTTPAPRPPTPAP

TIASQPLSLRPEACRPAAGGAVHTRGLDFACDIYIWAPLAGTCGVLLLSLVITLYCKRGRKKLLYIFKQPF

MRPVQTTQEEDGCSCRFPEEEEGGCELRVKFSRSADAPAYQQGQNQLYNELNLGRREEYDVLDKRRG

RDPEMGGKPRRKNPQEGLYNELQKDKMAEAYSEIGMKGERRRGKGHDGLYQGLSTATKDTYDALHM

QALPPRGSGATNFSLLKQAGDVEENPGPMNYPLTLEMDLENLEDLFWELDRLDNYNDTSLVENHLCPA

TEGPLMASFKAVFVPVAYSLIFLLGVIGNVLVLVILERHRQTRSSTETFLFHLAVADLLLVFILPFAVAEGS

VGWVLGTFLCKTVIALHKVNFYCSSLLLACIAVDRYLAIVHAVHAYRHRRLLSIHITCGTIWLVGFLLAL

PEILFAKVSQGHHNNSLPRCTFSQENQAETHAWFTSRFLYHVAGFLLPMLVMGWCYVGVVHRLRQAQ

RRPQRQKAVRVAILVTSIFFLCWSPYHIVIFLDTLARLKAVDNTCKLNGSLPVAITMCEFLGLAHCCLNP

MLYTFAGVKFRSDLSRLLTKLGCTGPASLCQLFPSWRRSSLSESENATSLTTFGSGEGRGSLLTCGDVEE

NPGPMRISKPHLRSISIQCYLCLLLNSHFLTEAGIHVFILGCFSAGLPKTEANWVNVISDLKKIEDLIQSM

HIDATLYTESDVHPSCKVTAMKCFLLELQVISLESGDASIHDTVENLIILANNSLSSNGNVTESGCKECEE

LEEKNIKEFLQSFVHIVQMFINTS

Figure S5: Supporting sequences. CAR_P2A_CXCR5_T2A_IL-15 construct protein sequence

Table S1: Summary of necropsy findings from animals with adverse outcomes during the DRAGA mouse study.

DRAGA Number	DRAGA Sex	Donor number and sex	Necropsy Time point	Symptoms
28	F	1 (M)	1 Week post-ART	>10% weight loss in one week<br>Anemia (pale blood)<br>Multifocal white spots on liver<br>Cyst on left ovary
30	F	1 (M)	4 Weeks post-infection	>10% weight loss in one week<br>Abnormal behavior: circling, lethargy
32	F	1 (M)	2 Weeks post-ART	>10% weight loss in one week<br>Anemia (pale blood)<br>Multifocal white spots on liver
33	F	1 (M)	20 Days post-treatment (PBS group)	>10% weight loss in one week<br>Abnormal behavior: circling, lethargy
35	F	1 (M)	4 weeks post-ART	Abnormal behavior: extreme lethargy, trouble standing<br>Anemia (pale ears and tail)<br>Splenomegaly with multifocal white spots<br>Hemorrhagic cyst on ovary taking up approx. 1/2 of the total abdominal cavity, blood and pus in surrounding tissue<br>Blood in cranial cavity
42	F	2 (M)	2 days post-ART interruption	Anemia (pale blood)<br>Blood in cranial cavity
